# The Corvids Literature Database—500 years of ornithological research from a crow’s perspective

**DOI:** 10.1093/database/bav122

**Published:** 2016-02-11

**Authors:** Gabriele Droege, Till Töpfer

**Affiliations:** 1 Botanic Garden and Botanical Museum Berlin-Dahlem, Freie Universität Berlin, Koenigin-Luise-Str. 6-8, Berlin 14195, Germany; 2 Section Ornithology, Zoological Research Museum Alexander Koenig, Centre for Taxonomy and Evolutionary Research, Adenauerallee 160, Bonn 53113, Germany

## Abstract

Corvids (Corvidae) play a major role in ornithological research. Because of their worldwide distribution, diversity and adaptiveness, they have been studied extensively. The aim of the Corvids Literature Database (CLD, http://www.corvids.de/cld) is to record all publications (citation format) on all extant and extinct Crows, Ravens, Jays and Magpies worldwide and tag them with specific keywords making them available for researchers worldwide. The self-maintained project started in 2006 and today comprises 8000 articles, spanning almost 500 years. The CLD covers publications from 164 countries, written in 36 languages and published by 8026 authors in 1503 journals (plus books, theses and other publications). Forty-nine percent of all records are available online as full-text documents or deposited in the physical CLD archive. The CLD contains 442 original corvid descriptions. Here, we present a metadata assessment of articles recorded in the CLD including a gap analysis and prospects for future research.

**Database URL:** http://www.corvids.de/cld

## Introduction

Corvids (Corvidae) form a well-known bird family within the Passeriformes comprising approximately 130 extant and 18 extinct species in 28–30 genera, depending on preferred species concept and classification. Regarding their feeding habits, morphology, body size, social behaviour and habitat preferences, they are one of the most diverse bird families. Some species have a wide distribution range, others are close to extinction or already extinct in the wild. Their common association with human settlements, their intelligence, diversity and worldwide distribution makes them ideal target species for thousands of scientific publications and provide an excellent example to illustrate trends and topics in ornithological publications spanning almost 500 years.

**Figure 1:**
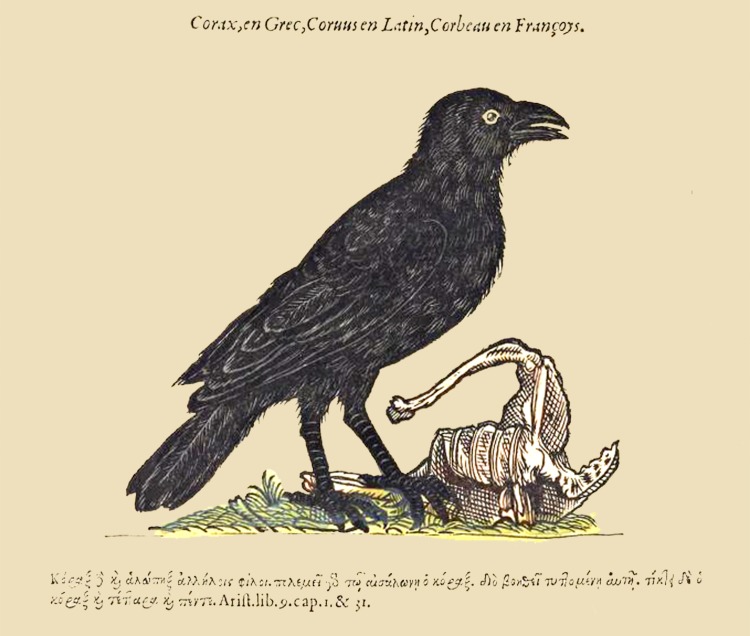
Illustration of a Raven in an ornithological book by Pierre Belon published 1555 in Paris (3). Note the common graphical presentation of ravens with bones of dead animals since the Middle Ages in Europe. Source contributed by Research Library, the Getty Research Institute; http://biodiversitylibrary.org/page/43989847.

Corvids play a major role in human culture. They are mythical, spiritual and religious symbols, especially in the Northern Hemisphere. Because of their extraordinary cognitive abilities, they are often associated with heavenly bodies and divinities, i.e. the Raven (*Corvus corax*) being the bird of Apollo and Odin. In many cultures, corvids and their shiny plumage are symbols for the sun and happiness (1), quite often also associated with the beginning of the world (2). For example, magpies are still symbols for happiness and joy in Eastern Asia. In Europe, the perception of ravens and crows was deeply influenced by the Middle Ages, associating them with death because of their scavenging habits, black plumage and sonorous voice (Figure 1).

The oldest known painting of a Raven is from Lascaux caves in France dated ca. 17 000 BC. Raven or crow-like drawings on pottery, weapons or stones are known from ancient Egypt, European antiquity in Greece and Italy as well as Scandinavia and Germany (6^th^–7^th^ century) (4). Later on corvids appear in many paintings in Asia, Europe and North America until today. In literature, corvids have often featured in tales and sagas for hundreds of years (2) and even appear in the Old Testament.

Scientific publishing in ornithology started in the 17^th^ century in Europe and at the end of 18^th^ century in North America (5). Today, handbooks and monographs such as Refs. 6–8 contain a lot of information but focus on European and American articles as references. Checking a list of 2000 publications about Rooks (*Corvus frugilegus*) for a diploma thesis (9) turned into a challenge. Generic topics on corvids are covered in depth, but detailed content on specific subjects, such as corvids as prey, is more difficult to find on the Internet in general.

To solve the problem mentioned above, Gabi Droege set up and developed a literature database about corvids that enables researchers from all over the world to search and contribute scientific publications related to these fascinating animals online. This includes journals published by various scientific societies as well as short notes, books, theses and field guides. Furthermore, semi-popular books and articles are included if they focus on corvids. From the start it was clear that the database would ultimately contain >20 000 articles, since corvids were study objects for thousands of researchers and appear in every report on distribution, breeding and ringing in regional ornithological journals worldwide. Here we present a metadata assessment of publications recorded in the Corvids Literature Database (CLD, http://www.corvids.de/cld), including a gap analysis and the outlook for planned focal points in the future. To demonstrate the importance of research on corvids for ornithology and biology in general, we take a closer look at the important gain of knowledge on cooperative breeding and tool use that was substantially fuelled by research on corvids.

## Materials and methods

At the beginning, main sources for CLD articles were EBSCO Biological Abstracts (http://www.ebscohost.com/academic/biological-abstracts), Zoological Record (http://wokinfo.com/products_tools/specialized/zr/) as well as reference lists in German monographs (10–13). Subsequently, other authors discovered the website and provided additional lists and articles. Furthermore, reference lists of each publication were checked as well as full journal volumes to find new records. A subscription to ∼150 journal alerts has been set up to become aware of recently published articles.

**Figure 2:**
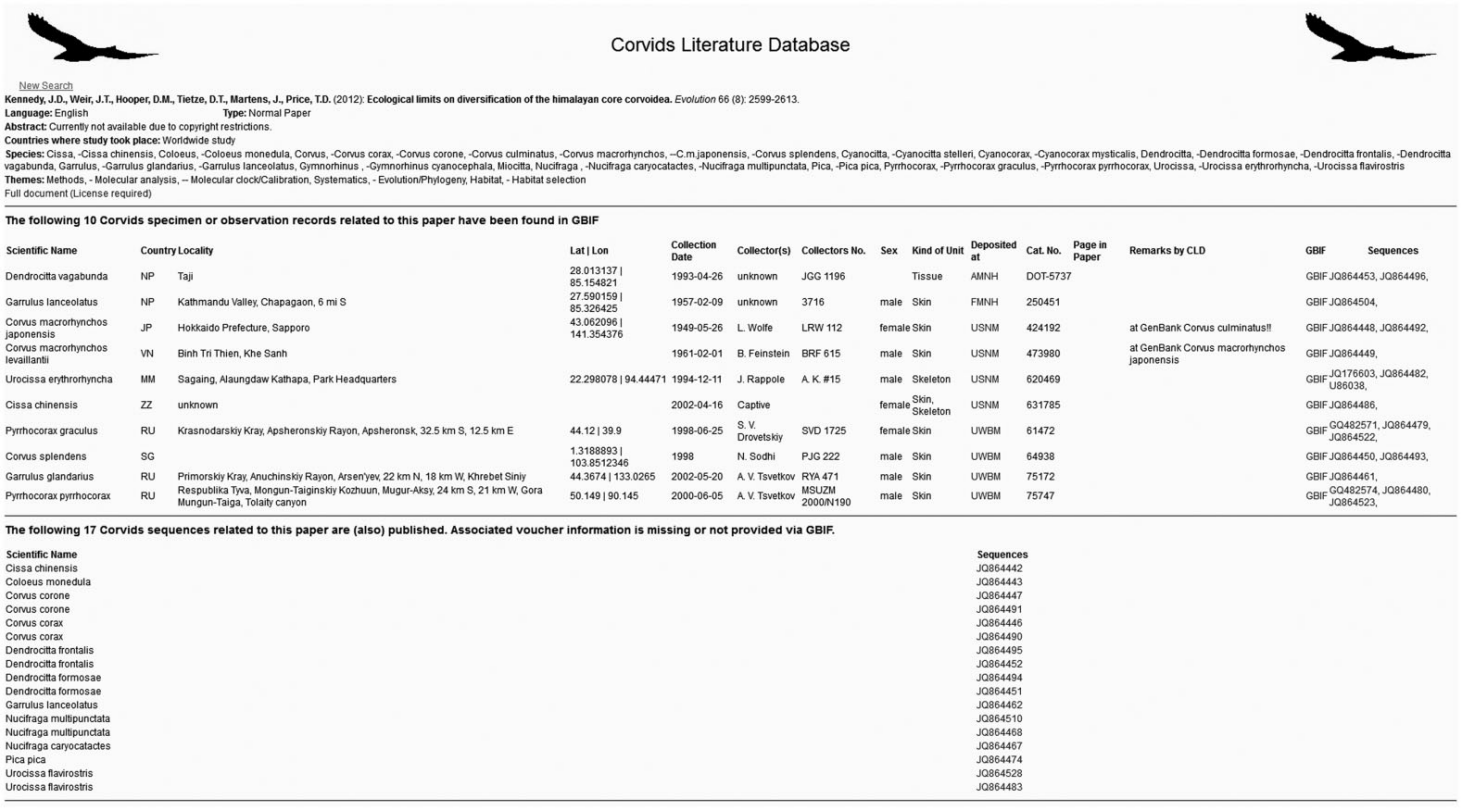
CLD entry with tagged topics, covered countries, taxa and language as well as additional information on corvid specimens and sequences related to this publication. http://www.corvids.de/cld/query.php?ID_Paper=6665.

Starting from those lists every single publication was checked for the following criteria: (i) corvid taxa covered, (ii) topics covered, (iii) language(s) of article, (iv) countries covered, (v) abstract and (vi) link to full document (Figure 2). Titles were recorded in the original language with an English translation if available. If digital copies are not available to retrieve, a physical copy was obtained. These copies are not shared via the Internet but stored in the physical CLD archive in Berlin. Because of copyright issues, abstracts are available with login only, but full text search within abstracts is possible without login. Links to full documents refer to non-profit scientific platforms such as Searchable Ornithological Research Archive (SORA, https://sora.unm.edu/) and Biodiversity Heritage Library [BHL, http://biodiversitylibrary.org/ (14)] or to relevant publishers’ pages if available. If the country or continent was not specified in the article, it was derived from the author’s affiliation, the taxa covered and other articles by this author. A publication was included in the CLD if a corvid was the main topic or if it was of some relevance, e.g. papers on parasites found in or on corvids or papers on corvids as an outgroup for molecular studies. CLD focused on scientific publications of national and international academic or ornithological societies, including scientific books as well as semi-popular articles and books if they were available as full documents or covered an interesting topic (e.g. importance of corvids in myths and arts). In addition to classical research papers or books, short notes (e.g. about a crow taking a snow-bath) as well as academic theses, field guides or breeding atlases were included in the CLD.

Updates were done at irregular intervals but frequently throughout the course of a year. The entry of one record took 2–4 min and was done manually. Getting full text information could take from a few seconds up to several days, including travelling to libraries in different cities (Berlin, Frankfurt, Bonn). Backups were carried out in tandem with every update. The CLD initially focused on European taxa and was subsequently extended internationally, specifically to taxa with fewer than 200 publications to increase the availability of published research about these rarely studied taxa.

We used Dickinson and Christidis (15) as a taxonomic basis for extant taxa. Original sources were checked before CLD followed recent changes therein. For 20 taxa, CLD follows other sources than (15). The CLD follows (16) and (17) and splits *Coloeus* (including Coloeus dauuricus and Coloeus monedula) from *Corvus* since their genetic distances indicate the isolation of the subgenus *Coloeus*. Furthermore, the CLD follows (16) and (18) and accepts *Corvus levaillantii* as well as Corvus culminatus currently as different species from Corvus macrorhynchos, since (18) found differences in morphology and call types of these three taxa. *Nucifraga multipunctata* is accepted by the CLD as a species following (16), since they have found morphological differences between *N. multipuncata* and *N. caryocatactes*. The CLD also follows Ridgely and Greenfield (19) and Bonaccorso *et al.* (20) and splits *Cyanocorax luxuosus* from *C. yncas* (including subspecies *centralis, confusus, glaucescens, maya, speciousus* and *vividus*). Bonaccorso *et al.* (20) have found distinct differences in habitat and social behaviour, as well as plumage and call type. *Cyanopica cooki* is accepted as species by CLD, following Fok et al. (21) who found that genetic divergence is basal in the phylogenetic tree of *Cyanopica*. *Cyanocorax yncas andicolus* is kept as a subspecies following Zimmer (22), who describes significant morphological differences between specimens from Columbia (*C. y. cyanodorsalis)* and Venezuela (*C. y. andicolus*). *Corvus macrorhynchos philippinus* is also kept as subspecies since erection to species level by Dickinson and Christidis (15) is suggested without explanation or references. *Cyanocorax hafferi* has been described by Cohn-Haft *et al.* (23) as a new species and is awaiting approval by the SACC (South American Classification Committee) as well as by the International Ornithological Committee. The CLD currently accepts its species status.

Further exceptions or changes might follow as more molecular population studies for other taxa become available. Hume’s Ground-Tit *Pseudopodoces humilis* is part of the CLD since it was ranked as a corvid until the beginning of 21^th^ century (24), likewise *Platylophus galericulatus* even though it is probably a shrike (25). Names of extinct taxa were taken from Refs. 26 and 27.

## Results

### Extent and temporal distribution of publications in the CLD

The CLD has been online since spring 2006 and currently holds 8000 publications, comprising 6271 papers, 758 short notes, 432 books, 108 ringing reports, 90 observation reports, 79 field guides, 71 dissertations, 44 diploma theses, 38 handbooks, 34 breeding atlases, 27 breeding reports, 24 monographs, 19 mixed reports and 5 miscellaneous documents. Of these, 3884 articles and books are present in the physical CLD archive. Therefore, 49% of all records are available as full text which is important for tagging the records. Moreover, 41% of all publications are linked to full documents available online and 29% of all publications have an abstract databased.

**Figure 3:**
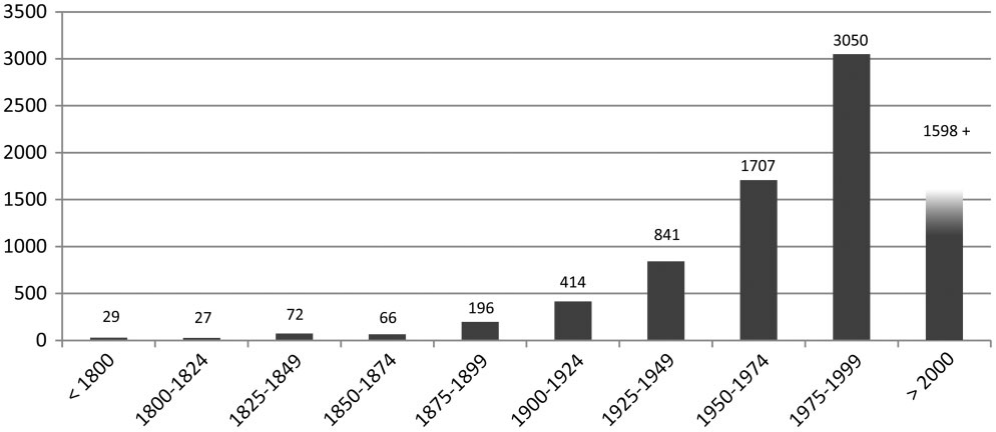
Number of articles in the CLD per 25 years. Note that the last column covers only 15 years.

The database spans almost 500 years of ornithological research, starting 1544 with Turner (28) (Figure 3).

Papers and notes are recorded from 1503 different journals. More than 100 corvid-related articles per journal are recorded from *British Birds* [201], *The Auk* [195], *Der Ornithologische Beobachter* [155], *Journal of Ornithology* [154], *The Ibis* [152], *Nos Oiseaux* [145], *Condor* [143], *Falke* [118] and *Die Vogelwelt* [103]. The only non-ornithological journal exceeding 100 records is *Animal Behaviour* [103 articles].

Articles are published in 36 languages, 53% in English, 27% in German, 5% in French and 3% in Russian. All other languages are represented by less than 1.2% each. Some articles have been published in more than one language.

The database holds records for 8026 different authors (persons and societies). Six authors have published >40 corvid-related articles: Manuel Soler [58], John M. Marzluff [47], Juan J. Soler [47], Glen E. Woolfenden [46], Bernd Heinrich [46] and Nicola S. Clayton [40]. A further 16 authors have published >20 articles each. These scientists had spent and are still spending large parts of their life on studying corvids and by this make a fundamental contribution to ornithological research in general. For example, Manuel and Juan J. Soler spent more than two decades studying co-evolution of Cuckoos parasitizing Magpies (*Pica pica*) and Hooded Crows (*C.* *corone cornix*) in Spain (29). Glen E. Woolfenden studied numerous aspects of the breeding biology of Florida Scrub Jays (*Aphelocoma coerulescens*) during his career (30) and John M. Marzluff studied many different topics related to corvids, such as learning, urbanization and anthropogenic influence (31). Bernd Heinrich is famous for his behavioural studies on Ravens (*C.* *corax*) in the wild in Maine/the USA (32), while Nicola S. Clayton studied learning and cognitive behaviour on different corvids in captivity (33).

**Figure 4:**
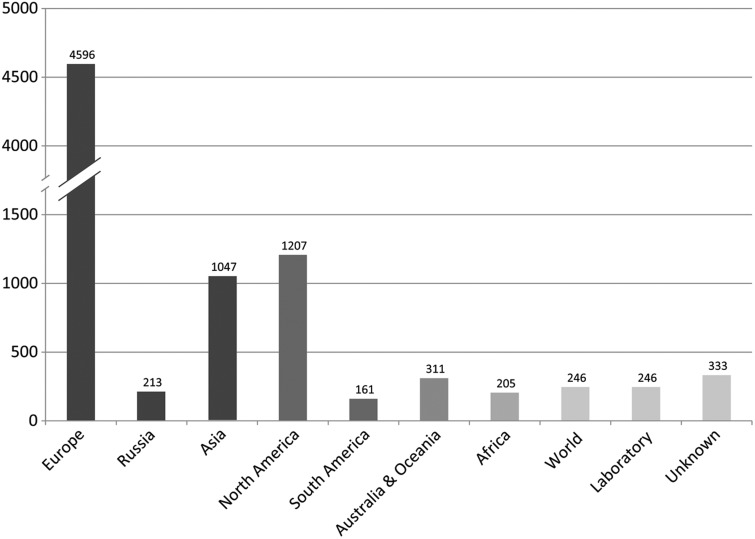
Numbers of articles per region recorded in the CLD. Individual countries are assigned to the respective geographical continent. Turkey is assigned to Asia. Russia is indicated separately due to its presence on two continents. ‘World’ refers to worldwide study foci.

Corvids were studied or collected in 164 countries and on all continents except Antarctica (Figure 4), which correlates with their worldwide distribution. Numerous publications comprise data from more than one country. Most studies took place in Europe, followed by North America and Asia. In total, 246 articles are based on captive birds (laboratory). For 333 articles, the country is unknown due to missing full text. Asia and Europe comprise same number of countries. Yet, in comparison, there are >100 articles recorded in the CLD for nine European countries each but only for three Asian countries plus Russia.

Altogether CLD currently holds 358 articles with 442 original corvid descriptions. Of these, 304 articles represent descriptions of all currently accepted extant and extinct taxa, including the family level. In total, 197 authors or author teams have described currently accepted corvid taxa. Most taxa were described by Linnaeus [28], followed by Bonaparte, Ridgway [19], Gould [11], Hartert [10], Vieillot, Vigors [9], Lesson [8] and Boddaert, Hume, Pitelka, S.F. Baird [7]. The most recent description of an extant taxon was published in 2013 (23). In addition, a lot of original names today considered synonyms are part of the database; however, searching for these names is not implemented yet but will be in the near future.

**Figure 5:**
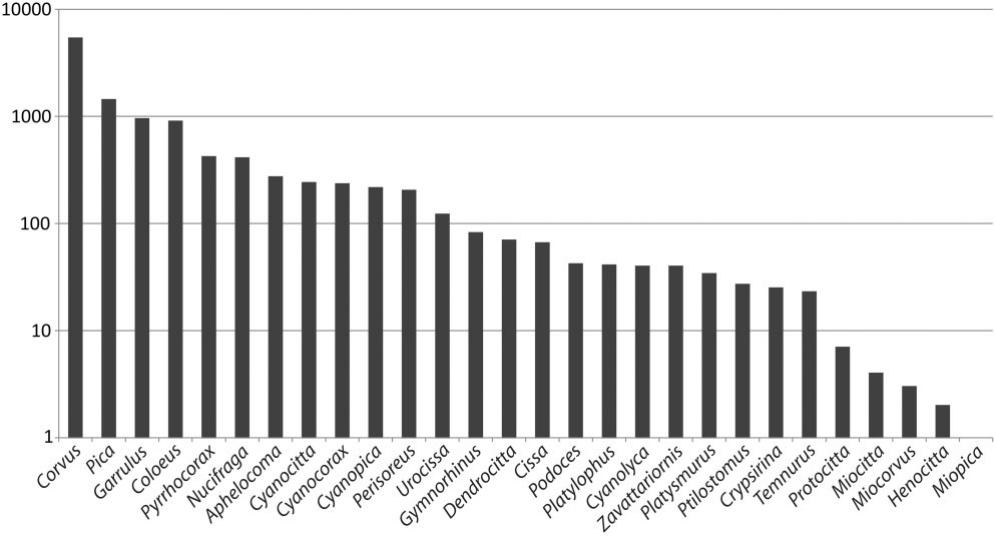
Number of articles per genus recorded at CLD. Note the logarithmic scale: more than two-thirds of the data solely refer to *Corvus*, 18% to *Pica*, 12% to *Garrulus* and 11% to *Coloeus*. Many articles cover more than one taxon.

Regarding the number of articles per genus for all genera, the largest proportion comprises articles on North American and European genera (Figure 5). Sixty-seven percent of all articles contain information about the largest genus *Corvus* (45 species). Among the articles with >200 records, except for *Corvus* and *Aphelocoma*, the genera comprise two or three species only. For other larger genera such as *Cyanocorax* (18 species) and the Asian genera *Cyanolyca* (9 species), *Dendrocitta* (7 species) and *Urocissa* (5 species) fewer than 125 publications are recorded, respectively.

Most articles are recorded for *C.* *frugilegus* [1943], followed by *C. corone* [1543], *P.* *pica* [1253], *C. corax* [1164], *Garrulus glandarius* [932], *Coloeus monedula* [861], *C.* *corone cornix* [572], *C. c. corone* [489], *Corvusbrachyrhynchos* [360], *Nucifraga caryocatactes* [342] and *C. splendens* [337]. An up-to-date list of all taxa counts can be found at http://www.corvids.de/cld/Status.php.

**Figure 6:**
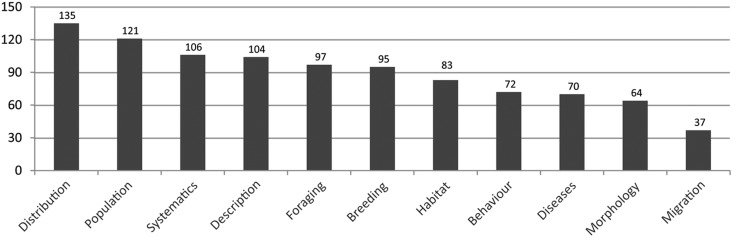
Number of countries per covered topic recorded at CLD. Only topics with >500 articles each are shown. Many articles cover more than one topic.

The CLD makes use of 246 different tags to allow topic-specific searches. Most of the articles cover distribution [2111], breeding [2049], foraging [2037], behaviour [1738], population [1431] and habitat [1404] as broader topics. Many topics are related to studies comprising >50 countries (Figure 6). In addition, articles related to systematics or descriptions also cover approximately 100 countries each.

Further examples of corvid-specific topics reported from several countries are brood parasitism (e.g. by cuckoos) (14 countries/86 articles), hybridization (15 countries/42 articles), corvids as prey (27 countries/156 articles), predation by corvids (39 countries/402 articles) and corvids on big herbivores (10 countries/34 articles).

**Figure 7:**
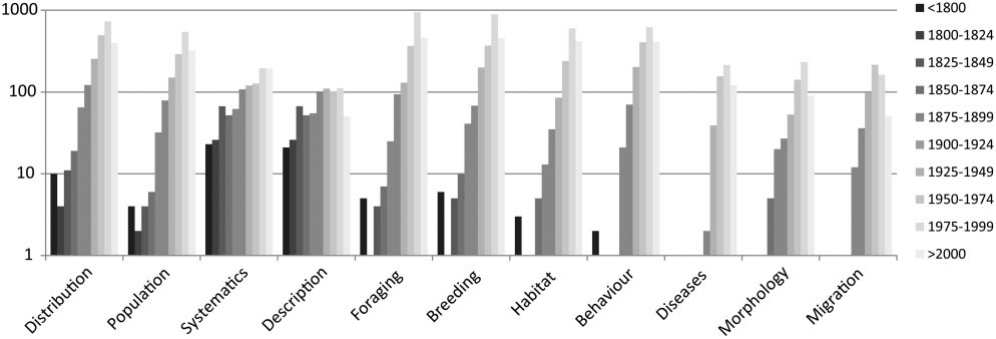
Number of articles for most common topics in CLD per 25 years. Note logarithmic scaling, only topics with >500 articles each are shown.

Regarding the temporal and topical aspects of corvid literature (Figure 7), before 1875 publications focused on systematics and descriptions only. Fewer than five articles each were published about behaviour, migration or morphology and articles on diseases are lacking. All other topics were published in fewer than 44 articles each. A strong increase in the number of articles is recorded from 1900 to 1924 for all topics, apart from diseases, morphology, migration and habitat. A strong increase can be seen from 1925 to 1949 for breeding, foraging, distribution, population, behaviour and migration. Migration is a subtopic of behaviour within CLD. From 1950, most published articles cover distribution, population, foraging, breeding and behaviour. Furthermore, articles on diseases, morphology and habitat increased substantially after 1950. The number of articles with taxon descriptions decreases from 1975, while systematics increases. Systematics as a CLD topic includes description and studies on evolution. Description includes original descriptions but also general taxonomic descriptions, e.g. found in monographs or in early expedition reports.

### Important topics covered by CLD

#### Molecular research

As early as 1967, corvids were already included in molecular research (34). A milestone in ornithological research and taxonomy was the publication of the Sibley–Ahlquist taxonomy in 1990 (35) providing the first comprehensive DNA-based taxonomy on birds, including corvids. Meanwhile, >1000 articles based on DNA data have been published within ornithology (36) since this ground breaking study. In the CLD, 210 molecular studies related to 100 corvid species are listed after 1990 with samples from 37 countries. Therefore, approximately 20% of all published molecular articles cover at least one corvid taxon. In 23 of these articles, corvids are used as outgroup representatives. The most recent mega project on whole genomes of selected bird species also includes a corvid (*C.* *brachyrhynchos*, ∼20 000 accession numbers) (37–39). Poelstra *et al.* (40) provided genomic data of *C.* *corone cornix* (∼29 000 accession numbers). This caused a 10-fold increase in sequence numbers for Corvidae at the International Nucleotide Sequence Database Collaboration (41) from 7250 to 70 774 (42). Since the last review in 2014, 21 new molecular studies on corvids have been published, including sequences of four further subspecies within *Corvus* as well as a comprehensive supermatrix phylogeny of corvoid passerines (43). Nevertheless, 50% of all extant taxa (primarily subspecies from Asia and Africa) and 100% of all extinct corvid taxa still lack molecular sequence data.

#### Cooperative breeding

The evolution of breeding systems is correlated to geographic and phylogenetic expansion (44). Cooperative breeding is quite common among Passeriformes and appears to have originated in Australia (45, 46). One of the first studies to shed light on this seemingly altruistic behaviour in birds was performed by Skutch in 1935 (47) who observed Brown Jays (*Cyanocorax morio*) in Central America. He discovered that offspring from the previous year help their parents raise the next generation and therefore this behaviour cannot be called altruistic. There is extensive research on Mexican Jays (*Aphelocoma ultramarina*) indicating kin selection (48–50) also known as Hamilton’s rule (51). Woolfenden and Fitzpatrick (30) carried out long-term research on the breeding biology of Florida Scrub Jay (*Aphelocoma coerulescens*), answering the question why some young help their parents and some do not. All of them made important contributions to the study of cooperative breeding in birds in general. The CLD holds 133 records for this topic, reported for 36 corvid species in 21 countries. In comparison with other large bird families (> 100 species), the percentage of species exhibiting cooperative breeding is much higher in corvids (46).

#### Tool use

Tool use by animals was first described by Wolfgang Köhler in 1917 (52) in captive chimpanzees and by Edward W. Gifford in 1919 (53) for birds. Since it is closely related to cognitive behaviour as well as forward planning and therefore deals with the old question of what distinguishes humans and animals, it was a very controversial topic for decades. The first observation of tool use by a corvid published in 1928 by Marc Le Goupils (54) for the New Caledonian Crow (*Corvus moneduloides*), describes how a crow uses a stick to catch insects from a hole in a tree. After a long period of silence, Jane Goodall published ground-breaking articles on tool use by wild chimpanzees (55, 56). Her study methods and results revolutionized ethology and showed that humans and chimpanzees have similar behaviour and that even waging wars is not only something that humans do. As a result of this and similar studies, cognitive intelligence in primates was accepted earlier than for birds. However, corvids played a major role in expanding this acceptance to birds: fundamental publications on the New Caledonian Crow and their tool use both in the wild and in captivity marked the turning point (57–60). Today it is no longer disputed that birds have the skills and anatomical requirements to allow complex cognitive behaviour and meanwhile tool-use is known to be much more widespread in birds than in mammals (61). The CLD holds 107 articles on tool use reported for 27 corvid species in 7 countries, most of which refer to the New Caledonian Crow (77 articles). Tool use has now been recorded for 12 species within one genus (*Corvus*), a striking total among birds in general. For comparison, Shumaker *et al.* (62) collected 700 references for tool use by great apes (Hominidae), comprising 6 species (excluding *Homo sapiens*).

#### Interoperabilities of CLD

CLD is steadily compiling a list of all known corvid synonyms to facilitate name matching of old literature and to enable searches for those names. This checklist will be provided to the Global Biodiversity Information Facility (GBIF, http://www.gbif.org/dataset/search?type=CHECKLIST) checklist bank when finished including concept relations and citing information on original descriptions. Lists of vernacular names already exist and can be fed into CLD using existing web services.

Other topics related to GBIF are specimen and observational records in the literature. CLD has started extracting such data from 30 articles so far, resulting in 1164 records. About 50% of them already exist in GBIF (specimens from natural history collections) and could be linked to the CLD. The other half mainly consist of observational records and are provided by the CLD to GBIF (http://www.gbif.org/dataset/829cf3b4-f762-11e1-a439-00145eb45e9a) using the BioCASe Provider Software (63). A meta-analysis of these data will be presented in a separate publication. Related parts of the CLD (specimen and observational records, Corvids of the World portal [EDIT Platform for Cybertaxonomy (64)], BioCASe, images) are kindly hosted by the Botanic Garden and Botanical Museum Berlin-Dahlem at Freie Universität Berlin.

## Discussion and perspectives

Beginning with Linnaeus’ *Systema Naturae* in 1758 (65), articles on systematics and descriptions were published in rapid succession including corvids until the late 19^th^ century. After the introduction of trinomials (names for taxa below the rank of species) (66, 67), the number of original descriptions increased enormously (68) which is reflected in the CLD as well. After 1975, the number of descriptions decreased while the number of articles on systematics kept increasing due to emerging molecular techniques. Articles on breeding, foraging, distribution, population studies and behaviour (including migration) appeared from 1875. The first four topics mentioned are represented, e.g. in the form of descriptions of egg collections and observations on nesting, foraging and occurrences in general. Research articles focusing on migration of birds appeared as early as the late 19^th^ century, with the first important contributions by Darwin in 1872 (69) and Wallace in 1874 (70). Starting from 1925, the advent of systematic field studies as a new method as well as extensive studies on captive birds led to another strong increase in the number of articles on breeding, foraging, distribution, population studies and behaviour. Articles on migration multiplied after 1950 caused by the worldwide establishment of banding programs and the use of radar systems (5). However, only a few North American and Eurasian corvids migrate regularly (8), hence the absolute number of articles on this topic is relatively low within the CLD. After 1950, articles on diseases and habitat increased, based on general rapid growth of cities worldwide and the start of globalization and the correlated consequences for human health.

Scientific ornithological publishing has a long tradition in Europe with numerous regional and local journals. Because of its European nucleus, the CLD currently contains a high percentage of German articles and articles about European taxa but relatively little on North American taxa. In general, non-English articles from Europe or Asia are more difficult to find on the Internet since only a few publishers enable online access. In addition, article titles do not always include the vernacular or scientific name of the taxon. A suitable way to receive further relevant articles from Europe and Asia in the future will be to include local researchers with complementary language skills.

Since the Raven (Corvus corax) has by far the widest distribution area (Eurasia, northern Africa, North America, Mexico), it is expected that most articles will be recorded for the Raven as soon as more North American literature has been added. The number of articles for American Crow (*C.* *brachyrhynchos*) and Black-billed Magpie (*Pica hudsonia*) will presumably be similar to that of their European equivalents Carrion Crow (*C. corone*) and Magpie (*P.* *pica*). The high number of articles about Rooks (*C.* *frugilegus*) shows how many publications exist but are almost impossible to find except by continuous and systematic trawling of reference lists and other sources constantly and systematically over years.

In contrast to methodological gaps, the data analysis shows that little is published about six of the seven monotypic genera as well as the Asian treepies (four genera) and oriental magpies (two genera). Including non-English Asian literature will increase the number of publications on treepies, but extensive research on their behaviour, distribution and evolution is nevertheless lacking. A detailed analysis of previously published data on this group is planned. Surprisingly, there are also no monographs on Choughs (*Pyrrhocorax*) or the Azure-Winged Magpie (*Cyanopica*) even though hundreds of articles have been published on these European genera. In contrast, monographs exist for every North American genus of corvids. The focus for upcoming records will therefore be on the extensive reference lists of North American and European non-English monographs to fill these methodological gaps in the CLD.

The temporal distribution of articles on corvids in the CLD follows the general trend in ornithological publication (5) although the enormous peak after the year 2000 is not visible in the CLD yet. Because the CLD’s main reference lists were taken from monographs published before 2000, a certain number of articles might still be missing. However, screening journal volumes for relevant articles published after 2000 will be done in the near future.

The inclusion of articles from before 1900 is much more difficult. Older synonyms have to be matched to currently accepted taxon names and the different style of writing complicates finding and tagging relevant articles. A milestone in digitization efforts was the launch of the BHL portal in 2007 (14), which represents an important source for the CLD (16% of all full text links). Screening BHL for relevant articles is possible, but cannot be automated since BHL search algorithm finds every scanned page where a name appears and not the article itself. BHL uses uBio name parser to allow full text search for taxon names, which includes many old synonyms and improves the BHL search a lot. CLD has not yet started to screen BHL but will do so in the near future.

Adding a particular record to the CLD cannot be automated without loss of information due to the granular management of topics and associated information. Therefore, CLD aims at automating detection of new (and very old) records in the Internet as well as automatically checking whether an article already exists in the CLD or not. The powerful tools provided by BHL (APIs), BioStor (citation parser, specimen catalogue number detection) and publishers (journal alerts, CrossRef) are very useful and thus will be adopted by CLD to include further literature in a more effective way.

Ornithological literature databases are mostly bibliographical lists. An exception is the Global Raptor Information Network (GRIN, http://www.globalraptors.org/grin/indexAlt.asp), which provides keyword search on literature and contains some corvid-related articles. In comparison, the CLD has a taxonomic (corvids), rather than an ecological scope and complements GRIN. The database OWL (Ornithological Worldwide Literature) contains >82 000 articles, but it is no longer maintained, database search is no longer possible and this valuable resource is therefore no longer available.

Because of their enormous diversity and adaptiveness, research on corvids will continue or even increase. A platform like CLD helps to find relevant articles that might be hard or impossible to trace otherwise. The maintenance and curation of CLD is expected to be continuous. Further contributors and editors are welcome to join the project and feedback will be much appreciated.

## References

[1] Werness H.B. (2003) Continuum Encyclopedia of Animal Symbolism in World Art. Continuum International Publishing Group Ltd London, NY.

[2] Feher-Elston C. (1991) Ravensong: A Natural and Fabulous History of Ravens and Crows. Northland, Flagstaff, AZ.

[3] Belon P. (1555) L'histoire De La Nature Des Oyseaux: Avec Leurs Descriptions, & NaïFs Portraits Retirez Du Naturel, Ecrite En Sept Livres. Gilles Corrozet, Paris.

[4] Bailleul-LeSuer R. (ed.) (2012) Between Heaven and Earth. Birds in Ancient Egypt. Oriental Institute Museum Publications 35. Chicago, IL.

[5] Birkhead T. Wimpenny J. Montgomerie B. (2014) Ten Thousand Birds: Ornithology since Darwin. Princeton University Press. Princeton, NJ.

[6] Goodwin D. (1976) Crows of the World. Cornell University Press, Ithaca, NY.

[7] Coombs C.J. (1978) The Crows. A Study of the Corvids of Europe. B. T. Batsford Ltd., London, UK.

[8] del Hoyo J. Elliott A. Christie D (ed). (2009) Handbook of the Birds of the World. Volume 14. Bush-Shrikes to Old World Sparrows. Lynx Edicions, Barcelona, Spain.

[9] Droege G. (2005) Freilandbiologische Untersuchung einer Brutkolonie der Saatkrähe *Corvus frugilegus*, L. in unmittelbarer Nachbarschaft zum Flughafen Berlin-Tegel. *Diploma Thesis.* Freie Universität Berlin.

[10] Gerber R. (1956) Die Saatkrähe. Neue Brehm-Bücherei (181). A. Ziemsen, Wittenberg-Lutherstadt, Germany.

[11] Bährmann U. (1968) Die Elster. Neue Brehm-Bücherei (393). A. Ziemsen, Wittenberg-Lutherstadt, Germany.

[12] Keve A. (1969) Der Eichelhäher. Neue Brehm-Bücherei (410). A. Ziemsen, Wittenberg-Lutherstadt, Germany.

[13] Glandt D. (2008) Der Kolkrabe—Der 'Schwarze Geselle' Kehrt Zurück. Aula-Verlag, Wiebelsheim, Germany.

[14] Gwinn N.E. Rinaldo C.A. (2009) The Biodiversity Heritage Library: sharing biodiversity with the world. IFLA Journal, 35, 25–34.

[15] Dickinson E.C. Christidis L. (ed.) (2014) The Howard and Moore Complete Checklist of the Birds of the World, 4th edn, Vol. 2. Passerines. Christopher Helm Publishers. London.

[16] Rasmussen P.C. Anderton J.C. (2012) Birds of South Asia. The Ripley Guide. Volume 2. 2nd edn Lynx Edicions, Barcelona, Spain.

[17] Kryukov A.P. Suzuki H. (2000) Phylogeography of carrion, hooded, and jungle crows (Aves, Corvidae) inferred from partial sequencing of the mitochondrial DNA cytochrome b gene. Russian J. Genet., 36, 922–929. 11033783

[18] Martens J. Böhner J. Hammerschmidt K. (2000) Calls of the Jungle Crow (*Corvus macrorhynchos* s.l.) as a taxonomic character. J. Ornithol., 141, 275–284.

[19] Ridgely R.S. Greenfield P.J. (2001) The Birds of Ecuador. Volume 1. Status, Distribution and Taxonomy. Cornell University Press. Ithaca, NY.

[20] Bonaccorso E. Peterson A.T. Navarro-Sigüenza A.G. (2010) Molecular systematics and evolution of the *Cyanocorax* jays. Mol. Phylogenet. Evol., 54, 897–909. 19931623 10.1016/j.ympev.2009.11.014

[21] Fok K.W. Wade C.M. Parkin D. (2002) Inferring the phylogeny of disjunct populations of the azure-winged magpie *Cyanopica cyanus* from mitochondrial control region sequences. Proc. R. Soc. Lond. [Biol.], 269, 1671–1679. 10.1098/rspb.2002.2057 PMC1691084 12204127

[22] Zimmer J.T. (1953) Studies of Peruvian birds. No. 65. The Jays (Corvidae) and Pipits (Motacillidae). *American Museum Novitates* no. 1649.

[23] Cohn-Haft M. Santos Junior M.A. Fernandes A.M. (2013) A new species of *Cyanocorax* jay from Savannas of the central Amazon In: del Hoyo J. Elliott A. Christie D. (ed). Handbook of the Birds of the World. New Species and Global Index, Special Volume, pp. 306–310. Lynx Edicions, Barcelona.

[24] James H.F. Ericson P.G.P. Slikas B. (2003) *Pseudopodoces humilis*, a misclassified terrestrial tit (Paridae) of the Tibetan Plateau: evolutionary consequences of shifting adaptive zones. The Ibis, 145, 185–202.

[25] Jønsson K.A. Irestedt M. Fuchs J. (2008) Explosive avian radiations and multi-directional dispersal across Wallacea: evidence from the Campephagidae and other Crown Corvida (Aves). Mol. Phylogenet. Evol., 47, 221–236. 18295511 10.1016/j.ympev.2008.01.017

[26] Brodkorb P. (1978) Catalogue of fossil birds. T. 5. Passeriformes. Bull. Fla. Museum Nat. Hist., 23, 139–228.

[27] Mlíkovský J. (2002) Cenozoic Birds of the World. Part 1: Europe. Ninox Press, Praha.

[28] Turner W. (1544) Avium Praecipuarum Quarum Apud Plinium Et Aristotelem Mentio Est, Brevis Et Succincta Historia. Gymnicus, Coloniae.

[29] Soler J.J. Martinez J.G. Soler M. (1999) Genetic and geographic variation in rejection behavior of cuckoo eggs by European magpie populations: an experimental test of rejecter-gene flow. Evolution, 53, 947–956. 10.1111/j.1558-5646.1999.tb05388.x 28565625

[30] Woolfenden G.E. Fitzpatrick J.W. (1984) The Florida Scrub Jay: Demography of a Cooperative-Breeding Bird. Princeton University Press, Princeton, NJ.

[31] Marzluff J.M. Angell T. (2012) Gifts of the Crow: How Perception, Emotion, and Thought Allow Smart Birds to Behave like Humans. Atria, NY.

[32] Heinrich B. (1989) Ravens in Winter. Simon and Schuster, NY.

[33] Legg E.W. Clayton N.S. (2014) Eurasian jays (*Garrulus glandarius*) conceal caches from onlookers. Anim. Cogn., 17, 1223–1226. 24638877 10.1007/s10071-014-0743-2 PMC4138428

[34] Sibley C.G. Brush A.H. (1967) The electrophoretic study of avian eye-lense proteins. The Auk, 84, 203–219.

[35] Sibley C.G. Ahlquist J.E. (1990) Phylogeny and Classification of the Birds of the World. Yale University Press, New Haven.

[36] Fjeldså J. (2013) Avian classification in flux In: del Hoyo J. Elliott A. Christie D. (ed.), Handbook of the Birds of the World. New Species and Global Index, Special Volume, pp. 77–146. Lynx Edicions, Barcelona.

[37] Jarvis E.D. Mirarab S. Aberer A.J. (2014) Whole-genome analyses resolve early branches in the tree of life of modern birds. Science, 346, 1320–1331. 25504713 10.1126/science.1253451 PMC4405904

[38] Zhang G. Li C. Li Q. (2014) Comparative genomics reveals insights into avian genome evolution and adaptation. Science, 346, 1311–1320. 25504712 10.1126/science.1251385 PMC4390078

[39] Zhou Q. Zhang J. Bachtrog D. (2014) Complex evolutionary trajectories of sex chromosomes across bird taxa. Science, 346, 1246338 25504727 10.1126/science.1246338 PMC6445272

[40] Poelstra J.W. Vijay N. Bossu C.M. (2014) The genomic landscape underlying phenotypic integrity in the face of gene flow in crows. Science, 344, 1410–1414. 24948738 10.1126/science.1253226

[41] Cochrane G. Karsch-Mizrachi I. Nakamura Y. (2011) The International Nucleotide Sequence Database Collaboration. Nucleic Acids Res., 39, D15–D18. 21106499 10.1093/nar/gkq1150 PMC3013722

[42] Droege G. Barker K. Astrin J. (2014) The Global Genome Biodiversity Network (GGBN) Data Portal. Nucleic Acids Res., 42, D607–D612. 24137012 10.1093/nar/gkt928 PMC3965106

[43] Jønsson K.A. Fabre P.H. Kennedy J.D. (2015) A supermatrix phylogeny of corvoid passerine birds (Aves: Corvides). Mol. Phylogenet. Evol., 94, 87–94. 26327328 10.1016/j.ympev.2015.08.020

[44] Marki P.Z. Fabre P.H. Jønsson K.A. (2015) Breeding system evolution influenced the geographic expansion and diversification of the core Corvoidea (Aves: Passeriformes). Evolution, 69, 1874–1924. 26095612 10.1111/evo.12695

[45] Arnold K.E. Owens I.P.F. (1998) Cooperative breeding in birds: a comparative test of the life history hypothesis. Proc. R. Soc. Lond. [Biol.], 265, 739–745.

[46] Cockburn A. (2006) Prevalence of different modes of parental care in birds. Proc. R. Soc. Lond. [Biol.], 273, 1375–1383. 10.1098/rspb.2005.3458 PMC1560291 16777726

[47] Skutch A.F. (1935) Helpers at the nest. The Auk, 52, 257–273.

[48] Brown J.L. (1970) Cooperative breeding and altruistic behaviour in the Mexican jay, *Aphelocoma ultramarina*. Anim. Behav., 18, 366–378.

[49] Brown J.L. (1972) Communal feeding of nestlings in the Mexican jay (*Aphelocoma ultramarina*): interflock comparisons. Anim. Behav., 20, 395–403.

[50] Brown J.L. (1987) Helping and Communal Breeding in Birds. Ecology and Evolution. Princeton University Press, Princeton, NJ.

[51] Hamilton W.D. (1970) Selfish and spiteful behaviour in an evolutionary model. Nature, 228, 1218–1220. 4395095 10.1038/2281218a0

[52] Köhler W. (1917) Intelligenzprüfungen an Anthropoiden I. Abhandlungen der Königlich Preußischen Akademie der Wissenschaften, 1917, Physikalisch-mathematische Klasse, Nr. 1.

[53] Gifford E.W. (1919) Field notes on the birds of the Galapagos Islands and of Cocos Island, Costa Rica. Proc. Calif. Acad. Sci., **Ser. 4**, 189–258.

[54] Le Goupils M. (1928) Dans La Brousse Calédonienne: Souvenirs D'un Ancien Planteur 1989-1904. Librairie Académique Perrin, Paris.

[55] Goodall J. (1964) Tool-using and aimed throwing in a community of free-living chimpanzees. Nature, 201, 1264–1266. 14151401 10.1038/2011264a0

[56] Van Lawick-Goodall J. (1968) The behaviour of free-living chimpanzees in the Gombe Stream Reserve. Anim. Behav. Monogr., 1, 161–311.

[57] Hunt G.R. (2000) Tool use by the New Caledonian Crow *Corvus moneduloides* to obtain *Cerambycidae* from dead wood. Emu, 100, 109–114.

[58] Hunt G.R. Corballis M.C. Gray R.D. (2001) Laterality in tool manufacture by crows. Nature, 414, 707 11742382 10.1038/414707a

[59] Chappell J. Kacelnik A. (2002) Tool selectivity in a non-mammal, the New Caledonian crow (*Corvus moneduloides*). Anim. Cogn., 5, 71–78. 12150038 10.1007/s10071-002-0130-2

[60] Weir A.A.S. Kenward B. Chappell J. (2004) Lateralization of tool use in New Caledonian crows (*Corvus moneduloides*). Proc. R. Soc. Lond. [Biol.], 271, 344–346. 10.1098/rsbl.2004.0183 PMC1810068 15504013

[61] Lefebvre L. (2013) Brains, innovations, tools and cultural transmission in birds, non-human primates, and fossil hominins. Front. Hum. Neurosci., 7, 245. 23761751 10.3389/fnhum.2013.00245 PMC3674321

[62] Shumaker R.W. Walkup K.R. Beck B.B. (2011) Animal Tool Behavior: The Use and Manufacture of Tools by Animals. Johns Hopkins University Press. Baltimore, MD.

[63] Holetschek J. Kelbert P. Müller A. (2009) International networking of large amounts of primary biodiversity data. Proceedings Informatik 2009—Im Focus das Leben. Lect. Notes Inform., 26, 552–564.

[64] Berendsohn W.G. Güntsch A. Hoffmann N. (2011) Biodiversity information platforms: from standards to interoperability. ZooKeys, 150, 71–87. 22207807 10.3897/zookeys.150.2166 PMC3234432

[65] Linnaeus C. (1758) Systema Naturae per Regna Tria Naturae, Secundum Classes, Ordines, Genera, Species, Cum Characteribus, Differentiis, Synonymis, Locis. Editio decima, Reformata Tomus I.

[66] Sharpe R.B. (1877) Catalogue of the Passeriformes or Perching Birds in the Collection of the British Museum. Coliomorphæ. Elibron Classics Series. British Museum, London.

[67] Seebohm H. (1882) Further notes on the ornithology of Siberia. The Ibis, 6, 419–428.

[68] Walters M. (2003) A Concise History of Ornithology. Christopher Helm, A & C Black, London.

[69] Darwin C. (1872) The Expression of the Emotions in Man and Animals. D. Appleton and Co., NY.

[70] Wallace A.R. (1874) Migration of birds. Nature, 10, 459

